# Transcription Factor p300 Regulated miR-451b Weakens the Cigarette Smoke Extract-Induced Cellular Stress by Targeting RhoA/ROCK2 Signaling

**DOI:** 10.1155/2022/7056283

**Published:** 2022-10-14

**Authors:** Wen Shen, Shukun Wang, Ruili Wang, Yang Zhang, Hong Tian, Xi Wang, Xin Wu, Xiaolei Yang, Wei Wei

**Affiliations:** Department of Respiratory Medicine, The Second Affiliated Hospital of Kunming Medical University, Kunming, China

## Abstract

**Background:**

A previous study identified miR-451b as a potential biomarker in smoker with or without chronic obstructive pulmonary disease (COPD). However, the function and molecular mechanisms of miR-451b in the pathogenesis of COPD remain elusive.

**Methods:**

Macrophages and lung fibroblasts were exposed to 10% cigarette smoke extract (CSE) solution for 24 h. Expression miR-451b and its potential transcription factor p300 were detected. The association between p300 and miR-451b, miR-451b and RhoA was validated by luciferase reporter assay. The release of IL-12 and TNF-*α*by macrophages was measured by ELISA assay, and Transwell assay was performed to analyze its migration and invasion. Collagen protein of fibroblasts was detected by Western blotting.

**Results:**

Results showed that p300 and miR-451b was downregulated, while RhoA was upregulated in CSE-induced macrophages and lung fibroblasts. The stimulation of CSE promoted the degradation of p300 by ubiquitination, and RhoA was confirmed as the target gene of miR-451b. MiR-451b overexpression significantly decreased the release of IL-12 and TNF-*α*, downregulated the expression of RhoA, ROCK2, and p65, and suppressed cell migration and invasion in CES-induced macrophages. In addition, miR-451b overexpression decreased the expression of RhoA, ROCK2, COL1A1, and COL2A1 in lung fibroblasts.

**Conclusions:**

Our data suggest that p300/miR-451b protects against CSE-induced cell stress possibly through downregulating RhoA/ROCK2 pathway.

## 1. Background

Chronic obstructive pulmonary disease (COPD) is widely considered an incurable but preventable respiratory disease with a rise in prevalence and mortality, which has been the third most frequent cause of death worldwide [1, 2]. The main symptoms of COPD include chronic cough, expectoration, emphysema, chronic airway obstruction, and airway remodeling [3]. Cigarette smoke extract (CSE) induced inflammatory disorder [4, 5] and impaired functional properties of lung fibroblasts [6, 7], are the important mechanisms underlying COPD. Although the association between CSE and COPD development has been reported [8–10], the cellular and molecular mechanism underlying COPD remains largely unclear.

MicroRNAs (miRNAs/miRs) are small non-coding RNA molecules that could regulate the transcriptional or translational gene expression via binding the 3′-untranslated region of multiple target mRNAs [11]. In recent years, some studies have shown that alterations in miRNA expression are closely associated with progression of smoking-induced patients with COPD [12, 13]. For example, miR-34a plays a key role in CSE-induced endothelial cell apoptosis by directly regulating its target gene Notch-1 [14]. Tang et al. [15] reported that miR-29b may participate in the airway inflammation in COPD by regulating inflammatory cytokine expression through targeting bromodomain protein 4 (BRD4). In addition, miR-146a is significantly downregulated in lung fibroblasts of COPD patients [16] and miR-26a acts as a regulator of the nuclear factor-*κ*B (NF-*κ*B) pathway in alveolar macrophages [17]. It is worth noting that our previous work identified several potential biomarkers, including miR-3202, miR-451b, and miR-149-3p in smokers with or without COPD and all their expression levels were downregulated in COPD group compared with control group [18]. In functional experiments, we demonstrated that reducing miR-149-3p may increase the inflammatory response in COPD patients through the regulation of the TLR-4/NF-*κ*B signaling pathway [18]. Similarly, our data provided support for the protective role of miR-3202 in CSE-stimulated T lymphocytes and human bronchial epithelial cells through targeting Fas apoptotic inhibitory molecule 2 (FAIM2) [19]. However, whether miR-451 plays an important role in suppressing CSE-induced lung injury has not been reported yet.

The small G-protein RhoA, as the master regulator of actin dynamics, is necessary for cell morphology, adhesion, proliferation, and migration [20]. Rho-kinase (ROCK-I or ROCK*β* and ROCK-II or ROCK*α*) is the downstream effector of RhoA and constitutes the RhoA/ROCK signal pathway involved in pulmonary endothelial dysfunction in healthy smokers [21] and patients with COPD [22]. In diabetic nephropathy, the RhoA/ROCK pathway might regulate NF-*κ*B activity to upregulate inflammatory genes [23]. In rheumatoid arthritis, blockade of ROCK inhibits the activation of NF-*κ*B and the production of pro-inflammatory cytokines [24]. Interestingly, CSE-induced p120-catenin- (p120-) mediated NF-*κ*B activation in human epithelial cells is dependent on the RhoA/ROCK pathway [25]. The online bioinformatics analysis suggests that RhoA was the target of miR-451b, which makes us hypothesize that the function of miR-451b in CSE-induced lung injury might through targeting RhoA/ROCK pathway.

To validate our hypothesis, CSE was first used to treat the macrophages and lung fibroblasts as useful *in vitro* models to evaluate smoking-related COPD pathogenesis. Then, we examined transcriptional regulation of miR-451b and its effects on inflammatory mediators, RhoA/ROCK pathway, extracellular matrix (ECM) components, migration, and invasion.

## 2. Materials and Methods

### 2.1. Sample Collection

Blood samples from non-smoking healthy volunteers (NS-H, *n* =10), smoking healthy volunteers (S-H, *n* =10), and smoking COPD patients (S-COPD, *n* =10) were collected from September 2019 to January 2020 at the Second Affiliated Hospital of Kunming Medical University. Total of 4 mL of peripheral blood was collected at fasting and half of it was used to isolate peripheral blood mononuclear cells (PBMCs) through gradient centrifugation with Ficoll-Hypaque (Ficoll-Paque PLUS; GE Healthcare Bio-Sciences AB, Uppsala, Sweden). The rest is used to separate serum.

### 2.2. Cell Culture

Macrophages (RAW264.7), rat pulmonary microvascular endothelial cells (rPMECs), rat alveolar epithelial cells (rAECs), rat bronchial epithelial cells (rBECs), and rat lung fibroblasts (rLFs) were purchased from Procell Life Science & Technology Co., Ltd. (Wuhan, China), which were both cultured in Dulbecco's Modified Eagle's Medium (DMEM) supplemented with 10% fetal bovine serum, 2 mM/L glutamine, 100 units/mL penicillin, and 0.1 mg/mL streptomycin at a humidified incubator containing 5% CO_2_ at 37°C.

### 2.3. Preparation of CSE

Preparation of CSE was performed using commercial cigarettes (Marlboro; Philip Morris USA, Richmond, VA, USA) by a modified method as previously described [26]. In brief, one cigarette was bubbled through 25 mL of DMEM at a constant rate, which was considered 100% CSE solution. Then, CSE solution was sterilized and diluted to a final working concentration (10%) before use.

### 2.4. Cell Transfection

MiR-451b mimics and inhibitor, specifically targeting RhoA small interference sequence, were synthesized by RiboBio (Guangzhou, China) (Table S1). Coding sequence was cloned and inserted into pcDNA 3.1 plasmid vector. For cell transfection, cells were seeded into six-well plates at a density of 3.0 × 10^7^ cells per well and cultured overnight at 37°C. Next day, cells were transfected with 25 nM above material for 48 h with Lipofectamine 2000 (Invitrogen, Carlsbad, CA), followed by 10% CSE exposure for an additional 48 h.

### 2.5. Quantitative Reverse Transcription PCR

Total RNA extraction was performed by Trizol reagent (Invitrogen) and cDNA was synthesized using a Reverse Transcription Kit (Applied Biosystems, Foster City, CA, USA) according to the manufacturer's instructions. Quantitative reverse transcription PCR was conducted with TaqMan Gene Expression Assays and the ABI Prism 7500 (Applied Biosystems) according to the thermal cycling conditions: 37°C for 10 min, 95°C for 5 min, followed by 50 cycles of 95°C for 15 s, 60°C for 20 s, and 68°C for 20 s. The primer sequences used in this study are showed in Table S1. Relative expression levels of miR-451b or RhoA were calculated by the 2^–*ΔΔ*Ct^ method with U6 or GAPDH as endogenous controls.

### 2.6. Western Blot Analysis

Total protein samples were extracted from cell samples with RIPA buffer (CWBio, Beijing, China) and protein concentration was measured using BCA protein assay kit (Beyotime, Shanghai, China). Equal amount of protein sample was separated on 12% SDS-PAGE gels followed by transferred to nitrocellulose membranes. Then, the membranes were blocked in 5% non-fat milk dissolved in TBST solution and incubated with primary antibodies against p300 (Abcam, Cambridge, MA, USA; ab275378, 1:1000 diluted), RhoA (ab187027, 1:2000 diluted), ROCK (ab134181, 1:1000 diluted), NF-*κ*B p65(ab207297, 1:1000 diluted), HDAC1 (ab109411, 1:4000 diluted), COL1A1 (ab270993, 1:3000 diluted), COL2A1 (ab34712, 1:3000 diluted), and GAPDH (ab8245, 1:5000 diluted) overnight at 4°C. After washing with TBST, membranes were incubated with HRP-conjugated secondary antibodies for 2 h. The protein bands were visualized using an enhanced chemiluminescence reagent (Pierce Biotech, Inc., Rockford, IL, USA).

### 2.7. Immunofluorescence

Cells from different groups were fixed with 4% paraformaldehyde, washed with PBS, and permeabilized with 0.5% Triton X-100 dissolved in PBS. After blocked with 3% bovine serum albumin (BSA) for 1 h, the cells were incubated with primary antibodies against RhoA (ab187027, 1:500 diluted) or p300 (ab275378, 1:500 diluted) overnight at 4°C. Subsequently, cells were washed with PBS twice and incubated with appropriate secondary antibodies. The nucleus was labeled with 4′,6-diamidino-2-phenylindole for 5 min. All staining images were viewed under a fluorescence microscope (Thermo Fisher Scientific, Waltham, MA, USA).

### 2.8. Enzyme-Linked Immunosorbent Assay (ELISA)

The release concentration of IL-12 and TNF-*α* was measured in cell culture media by commercial ELISA according to the manufacturer's instructions (R&D Systems) according to the manufacturer's protocol. All samples were assayed in duplicate. Results are expressed as picograms of cytokine per milligram (pg/mL) of total protein in the homogenate.

### 2.9. Transwell Assay

The migrated and invasive ability of macrophages was assessed using Transwell chamber (Corning Inc., Corning, NY, USA). In brief, approximately macrophages prepared in serum-free medium were added into the upper chamber (normal chamber for migration assay and matrigel-coated chamber for invasion assay). Meanwhile, complete medium (500 *μ*L) containing 10% FBS was added into the lower chamber. After 24 h incubation at 37°C, the macrophages that migrated into the lower chamber were fixed with methanol and stained with crystal violet, which were further counted with a microscope.

### 2.10. Luciferase Reporter Assay

Briefly, the oligonucleotides containing wild-type or mutated RhoA-3′UTR of the predicted binding site were synthesized and subcloned into psiCHECK-2 vector (Promega, Madison, Wisconsin) to construct WT or MUT RhoA plasmids, respectively. Then, co-transfection of WT or MUT RhoA plasmid and miR-451b mimics or NC was performed in RAW264.7 cells using Lipofectamine 2000. After 48 h, luciferase activity was determined using the dual-luciferase reporter assay system (Promega). Relative luciferase activity was reported as luciferase activity/Renilla luciferase activity. In addition, the 5′-upstream sequence of pre-miR-451b was segmented, cloned, and inserted into pGL3-Basic plasmid, followed by transfecting to RAW264.7 cells and fluorescence detection. To test and verify p300 regulating transcription of pre-miR-451b, the binding site sequence (5′-TTAGGGACTGAGTCT-3′) was mutated (5′-TTAATGCGGGAGTCT-3) and used to repeat fluorescence detection.

### 2.11. Chromatin Immunoprecipitation

Chromatin immunoprecipitation was performed according to the instruction of a ChIP Assay Kit (Beyotime). In brief, cells were lysed with ice-treated SDS lysis buffer and ultrasonication, and deoxyribonucleic acid (DNA) was extracted. Then, the sample was treated with ChIP dilution buffer and incubated with primary antibody targeted to p300. Subsequently, protein A agarose/salmon sperm DNA was added to precipitate the immune complex. Finally, wash the sediment, de crosslink, and recover DNA fragments. The 5′-upstream sequence of pre-miR-451b was detected by real-time quantitative PCR.

### 2.12. Electrophoretic Mobility Shift Assays

Promoter of pre-miR-451b-p300 banding was detected by probes that has been Biotin-labeled in the presence or absence of anti-p300 antibody. Prokaryotic expressed p300 protein was purified by gel filtration chromatography, followed by incubation with DNA probes in binding buffer. Anti-p300 antibody or specific mutant competitors were pre-incubated with p300. Finally, 4% PAGE gel was used to finish the electrophoretic separation to products.

### 2.13. Statistical Analysis

Each experiment was performed in triplicates and data were expressed as mean ± SD. Statistical analyses were carried out with GraphPad Prism version 6.0 (GraphPad Software, San Diego, CA, USA). Different comparisons were performed by Student's *t* test between two groups and one-way analysis of variances (ANOVA) followed by Tukey's test for three groups. Statistical significance was accepted when the *P*-value less than 0.05.

## 3. Results

### 3.1. Smoking Inhibits the miR-451b Expression through Regulating p300

**Figure 1:**
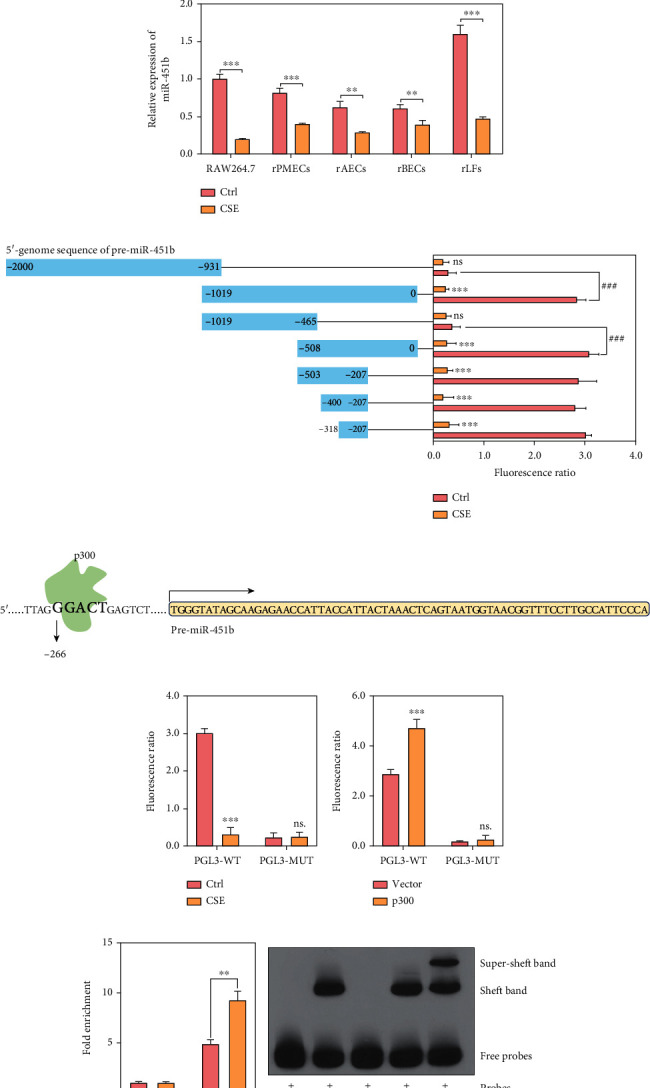
CSE inhibited expression of miR-451b through regulating RhoA. (a) Blood samples from non-smoking healthy volunteers (NS-H), smoking healthy volunteers (S-H), and smoking COPD patients (S-COPD) were collected and the expressions of miR-451b were detected by QPCR. ^∗∗∗^*P* < 0.001, compared with NS-H group. (b) Macrophages (RAW264.7), rat pulmonary microvascular endothelial cells (rPMECs), rat alveolar epithelial cells (rAECs), rat bronchial epithelial cells (rBECs), and rat lung fibroblasts (rLFs) were stimulated with 10% CSE, and the expressions of miR-451b were detected by QPCR. (c) The 5′-upstream sequence of pre-miR-451b was segmented, cloned, and inserted into pGL3-Basic plasmid, followed by transfecting to RAW264.7 cells and fluorescence detection. ^∗∗∗^*P* < 0.001, compared with Ctrl group (no CSE treatment). (d) Predicted binding site of p300 on miR-451b's promoter sequence. (e) The binding site of p300 was mutated and applied to luciferase reporter assay. (f) Luciferase reporter assay was repeated in p300 overexpressed RAW264.7 cells. (g, h) Chromatin Immunoprecipitation assay and Electrophoretic Mobility Shift assay were performed in p300 overexpressed RAW264.7 cells. ^∗∗^*P* < 0.01, ^∗∗∗^*P* < 0.001; ^###^*P* <0.001; ns: no significance.

To verify whether miR-451b is related to smoking, blood samples from non-smoking healthy volunteers (NS-H), smoking healthy volunteers (S-H), and smoking COPD patients (S-COPD) were collected and the expressions of miR-451b in which were detected. Results showed that the expression of miR-451b was significantly downregulated in serum and peripheral mononuclear cells (PBMCs) from both S-H group and S-COPD group, when compared with that from NS-H group (Figure 1(a)). Meanwhile, *in vitro* 10% CSE inhibited the expression of miR-451b in macrophages (RAW264.7), rat pulmonary microvascular endothelial cells (rPMECs), rat alveolar epithelial cells (rAECs), rat bronchial epithelial cells (rBECs), and rat lung fibroblasts (rLFs) (Figure 1(b)). Further, fluorescence report experiment in RAW264.7 showed that the core promoter sequence of miR-451b responding to CSE was region from -318 site ~ -207 site (Figure 1(c)). Through ALGGEN-PROMO database (http://alggen.lsi.upc.es/cgi-bin/promo_v3/promo/promoinit.cgi?dirDB=TF_8.3), the transcription factor, p300, could bind to the core promoter sequence of miR-451b (Figure 1(d)), and fluorescence report showed that there was no significant difference in fluorescence ratio between with or without CSE stimulation, when we mutated banding site of p300 in the core promoter sequence of miR-451b (Figure 1(e)). In addition, we overexpressed p300 in RAW264.7 cells, and fluorescence report showed that the fluorescence ratio was significantly increased (Figure 1(f)). Furthermore, the combined relationship between p300 and the core promoter sequence of miR-451b was confirmed by Chromatin Immunoprecipitation assay (Figure 1(g)) and Electrophoretic Mobility Shift assay (Figure 1(h)). Above results revealed that CSE may inhibited the expression of miR-451b in respiratory system related cells by regulating p300.

### 3.2. CSE Promoted Degradation of p300 by Ubiquitination Pathway

**Figure 2:**
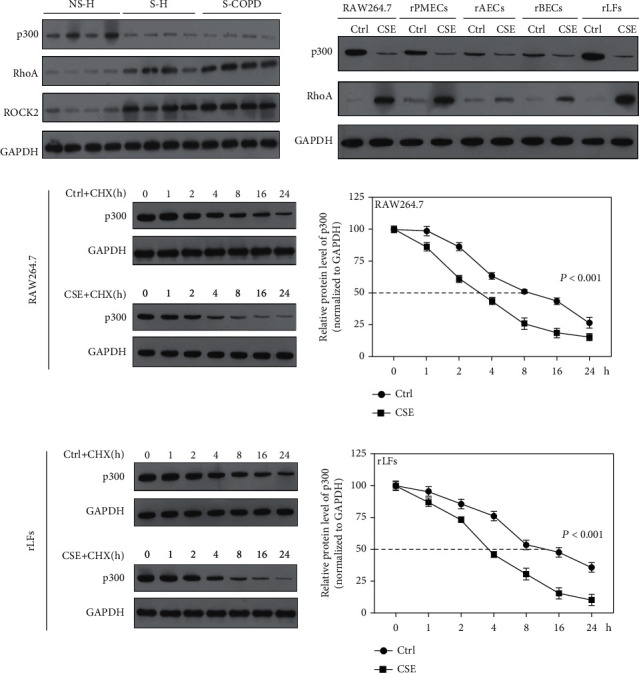
CSE induced the degradation of p300. (a) Western blotting was used to detect p300, RhoA, and ROCK2 in PBMCs. (b) Levels of p300 and RhoAin the CSE-stimulated RAW264.7, rPMECs, rAECs, rBECs, and rLFs. (c, d) The level of p300 at 0 h, 1 h, 2 h, 4 h, 8 h, 16 h, and 24 h after stimulation with CSE and cycloheximide (CHX) (100 *μ*g/mL) in RAW264.7 and rLFs was measured. NS-H: non-smoking healthy volunteers; S-H: smoking healthy volunteers; S-COPD: smoking COPD patients; Ctrl: no CSE treatment.

To explore the mechanism of miR-451b, we further detected the levels of p300 and miR-451b's potential downstream proteins, RhoA and ROCK2 in above PBMCs. Results showed that p300 in S-H and S-COPD group was decreased when compared with NS-H group, while RhoA and ROCK2 were increased in PBMCs derived from smokers (Figure 2(a)). In CSE-stimulated RAW264.7, rPMECs, rAECs, rBECs, and rLFs, we found that the protein levels of p300 were inhibited, and levels of RhoA were increased (Figure 2(b)). Further, we stimulated RAW264.7 and rLFs with CSE and cycloheximide (CHX), and cells were collected 0 h, 1 h, 2 h, 4 h, 8 h, 16 h, and 24 h after stimulation, followed by Western blotting to p300. Results showed that CSE stimulation significantly reduced the half-life of protein p300 (Figures 2(c) and 2(d)).

### 3.3. MiR-451b Targeted the 3′-UTR Regions of RhoA mRNA

**Figure 3:**
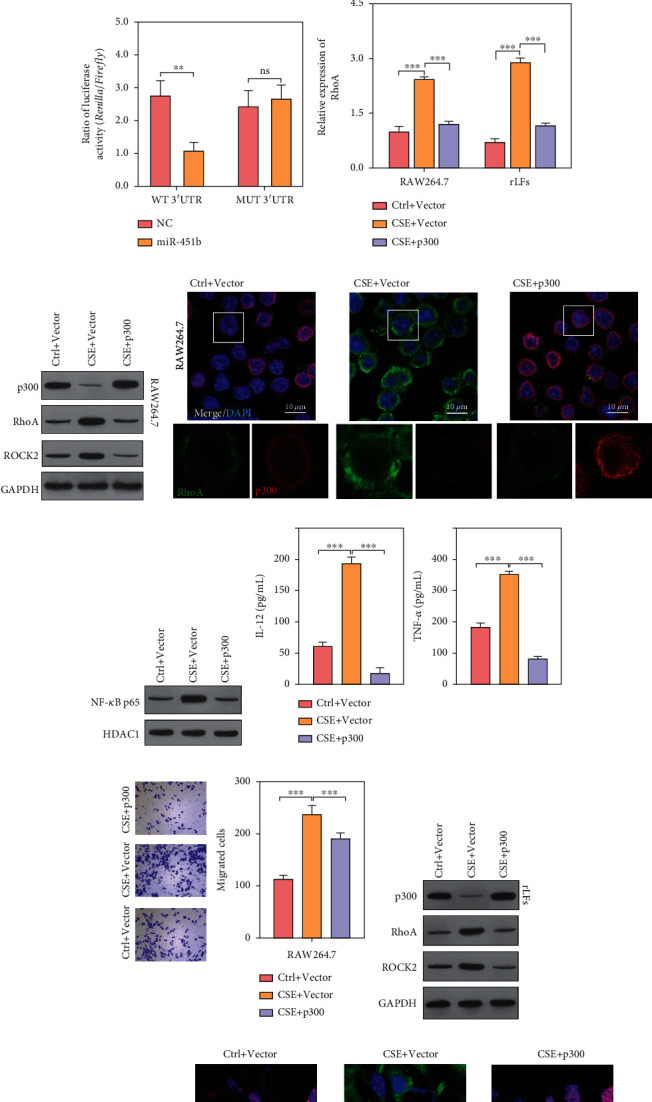
MiR-451b targeted the 3′-UTR regions of RhoA mRNA. The p300 was overexpressed in CSE-stimulated RAW264.7 and rLFs. (a) Expression of miR-451b was detected by QPCR. (b) Bioinformatics analysis shows that miR-451b binds to 3′-UTR regions of RhoA through base-complementary pairing. (c) RAW264.7 cells were co-transfected with psi-CHECK2-RhoA-3′-UTR (WT or MUT) and miR-451b mimics (or NC). After 48 h, luciferase activity was calculated as the ratio of Renilla luciferase activity to Firefly luciferase. (d) Expression of RhoA was detected by QPCR. (e) Western blot analysis was performed to analyze the protein of p300, RhoA, and ROCK2 in RAW264.7. (f) Protein level of p300 and RhoA in RAW264.7 was showed by immunofluorescence assay. (g) Nuclear NF-*κ*B p65 (normalized to HDAC1) in RAW264.7 was detected by Western blotting. (h) The pro-inflammatory cytokine IL-12 and TNF-*α* levels were detected by ELISA assay. (i) Cell migration was assessed in RAW264.7 by Transwell assay. (j, k) Protein of p300, RhoA, ROCK2, COL2A1, and COL1A1 in rLFs was showed by Western blotting. (l) Protein level of p300 and RhoA in rLFs was showed by immunofluorescence assay. ^∗∗^*P* < 0.01, ^∗∗∗^*P* < 0.001. NC: negative control. Ctrl: no CSE treatment; CSE+ p300: CSE treatment plus p300-overexpression; DAPI: 4′,6-diamidino-2-phenylindole.

In p300-overexpressed RAW264.7 and rLFs, we found that the decreased expressions of miR-451b induced by CSE stimulation were reversed (Figure 3(a)). Then, the online bioinformatics software was used to predict downstream target gene of miR-451b. As shown in Figure 3(b), miR-451b could bind to 3′-UTR regions of RhoA gene, which has been associated with pulmonary endothelial dysfunction in patients with COPD [22]. Subsequently, we performed luciferase report assay to confirm the association between miR-451b and RhoA. The results (Figure 3(c)) showed that miR-451b mimics transfection significantly suppressed the relative luciferase activity of the WT RhoA 3′-UTR compared with NC transfection in RAW264.7cells. In contrast, co-transfection of miR-451b mimics did not affect the luciferase activity of the binding site mutant RhoA 3′-UTR reporter. Further, we found that the expression of RhoA mRNA and protein was markedly increased by CSE stimulation, while it was reverted to low level by overexpression of p300 in RAW264.7 and rLFs (Figures 3(d)–3(f), 3(j), and 3(l)). Meanwhile, the level of ROCK2 and nuclear NF-*κ*B p65 was consistent with that of RhoA in RAW264.7 (Figures 3(e) and 3(g)), as well as the level of interleukin 12 (IL-12) and tumor necrosis factor-*α* (TNF-*α*) in culture supernatant (Figure 3(h)). Furthermore, CSE stimulation induced increasing migration ability of RAW264.7, and accumulation of proteins COL1A1 and COL2A1 in rLFs was both suppressed by overexpression of p300 (Figure 3(i)). These data suggest that RhoA might be a downstream target of miR-451b.

### 3.4. Overexpression of miR-451b and Knockdown of RhoA Inhibited Cell Migration and Inflammatory Factor Release in RAW264.7 Cells

**Figure 4:**
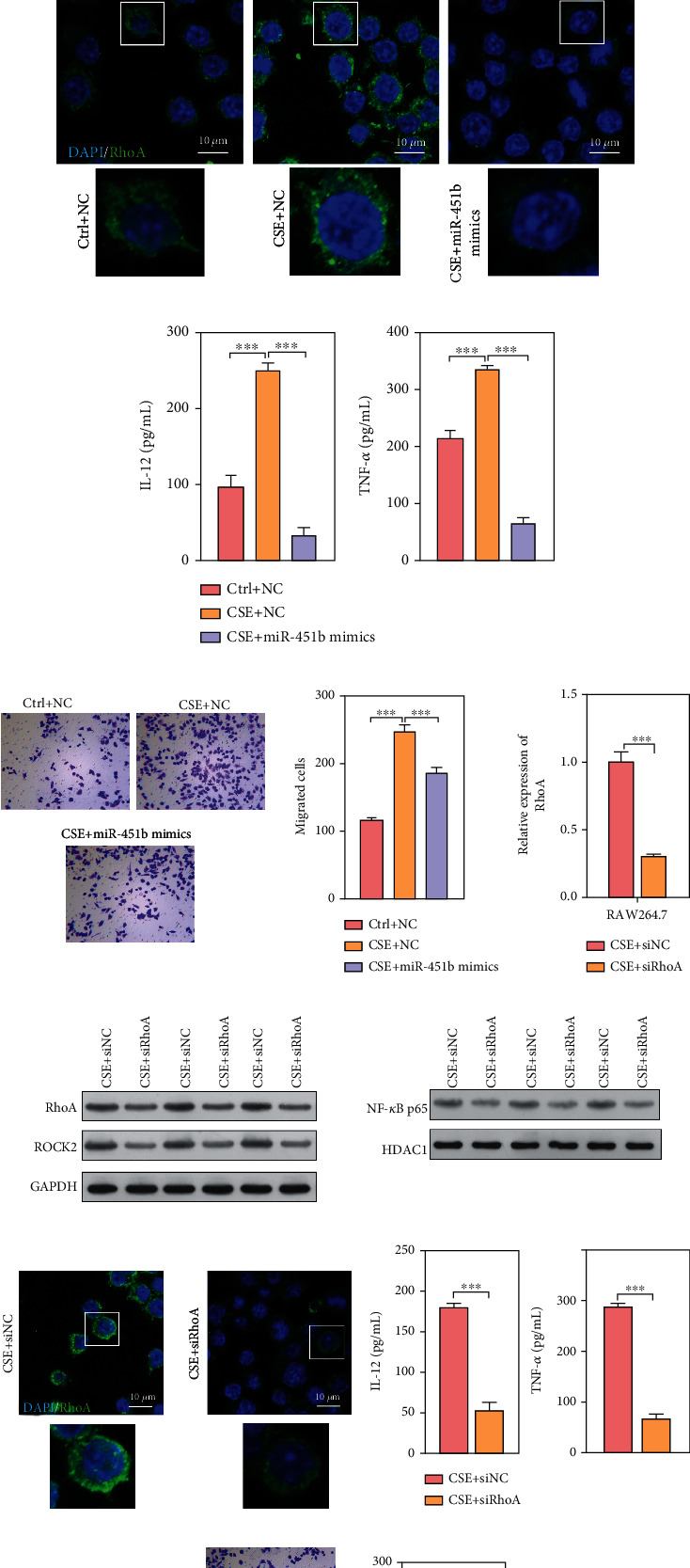
Overexpression of miR-451b or knockdown of RhoA decreased the effect of CSE on macrophages. RAW264.7 was transfected with miR-451b mimics or specifically targeting RhoA small interference sequence, followed by 10% CSE exposure. (a, f) The expression of RhoA mRNA was determined by QPCR. (b) Western blot analysis was performed to analyze the protein expression of RhoA, ROCK2, and nuclear NF-*κ*Bp65. (c, h) Immunofluorescence staining demonstrated the labeling intensity of RhoA. (d, i) The pro-inflammatory cytokine IL-12 and TNF-*α* levels were detected by ELISA assay. (e, g) Cell migration was assessed by Transwell assay. ^∗∗∗^*P* < 0.001. NC: negative control. Ctrl: no CSE treatment; CSE+ siRhoA: CSE treatment plus RhoA knockdown; DAPI: 4′,6-diamidino-2-phenylindole.

Since miR-451b was downregulated in RAW264.7 after CSE exposure, miR-451b mimics was transfected into RAW264.7 to investigate its impact on CSE-induced injury. At first, the expression of RhoA mRNA was demonstrated to be significantly upregulated in CSE exposure, which was notably decreased after miR-451b mimics transfection using quantitative reverse transcription PCR (Figure 4(a)). Results of Western blot analysis showed that miR-451b overexpression obviously downregulated the expression of RhoA, ROCK2, and nuclear NF-*κ*Bp65 in CSE-treated macrophages (Figure 4(b)). The immunofluorescence staining of RhoA (Figure 4(c)) also revealed similar result. Subsequently, the IL-12 and TNF-*α* levels (Figure 4(d)) were significantly decreased after miR-451b mimics transfection in CSE-treated RAW264.7, and the increased migratory cells in CSE group were remarkedly reduced after miR-451b overexpression (Figure 4(e)). In addition, we knockdown RhoA in CSE-stimulated RAW264.7 cells and results showed that RhoA mRNA and protein were both decreased (Figures 4(f)–4(h)). The protein level of ROCK2, concentration of IL-12 and TNF-*α* in culture supernatant, and cell migration ability were also suppressed by knockdown of RhoA (Figures 4(g), 4(i), and 4(j)).

### 3.5. Overexpression of miR-451b and Knockdown of RhoA Decreased Extracellular Collagen Accumulation in rLFs

**Figure 5:**
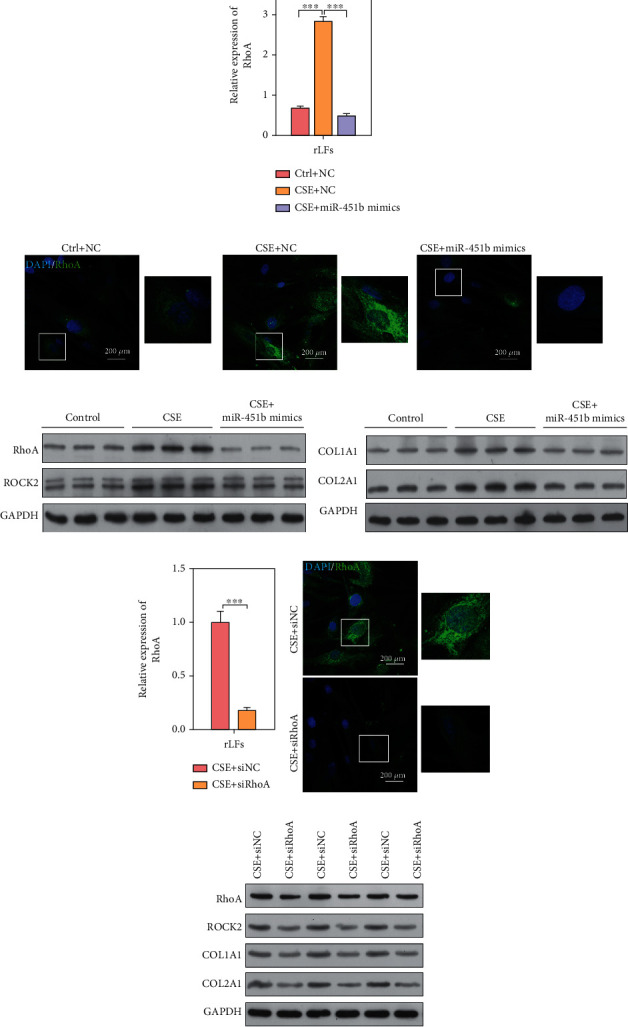
Overexpression of miR-451b or knockdown of RhoA decreased the effect of CSE on lung fibroblasts. The rLFs were transfected with miR-451b mimics or specifically targeting RhoA small interference sequence, followed by 10% CSE exposure. (a, e) The expression of RhoA mRNA was determined by QPCR. (b, f) Immunofluorescence staining demonstrated the labeling intensity of RhoA. (c, d, g) Western blot analysis was performed to analyze the protein expression of RhoA, ROCK2, COL1A1, and COL2A1. ^∗∗∗^*P* < 0.001. NC: negative control. Ctrl: no CSE treatment; CSE+ siRhoA: CSE treatment plus RhoA-knockdown; DAPI: 4′,6-diamidino-2-phenylindole.

Similarly, rLFs were transfected with miR-451b mimics and siRNA-specific targeting RhoA, followed by 10% CSE exposure. As shown in Figures 5(a) and 5(e), CSE-induced upregulation of RhoA mRNA in rLFs was significantly reduced after miR-451b mimics or siRhoA transfection. Consistently, immunofluorescence staining of RhoA further confirmed that upregulation of RhoA in CSE treatment was obviously impaired in rLFs after miR-451b mimics r siRhoA transfection (Figures 5(b) and 5(f)). What's more, we observed the obviously elevated protein expression of RhoA and ROCK2 induced by CSE was abolished by miR-451b mimics or siRhoA transfection (Figures 5(c) and 5(g)). We further found that accumulation of extracellular matrix (ECM) components (COL1A1 and COL2A1) in CSE group was significantly attenuated after miR-451b overexpression and knockdown of RhoA in rLFs (Figures 5(d) and 5(g)).

### 3.6. Knockdown of miR-451b Activated RhoA/ROCK2 Signaling in RAW264.7 and rLFs

**Figure 6:**
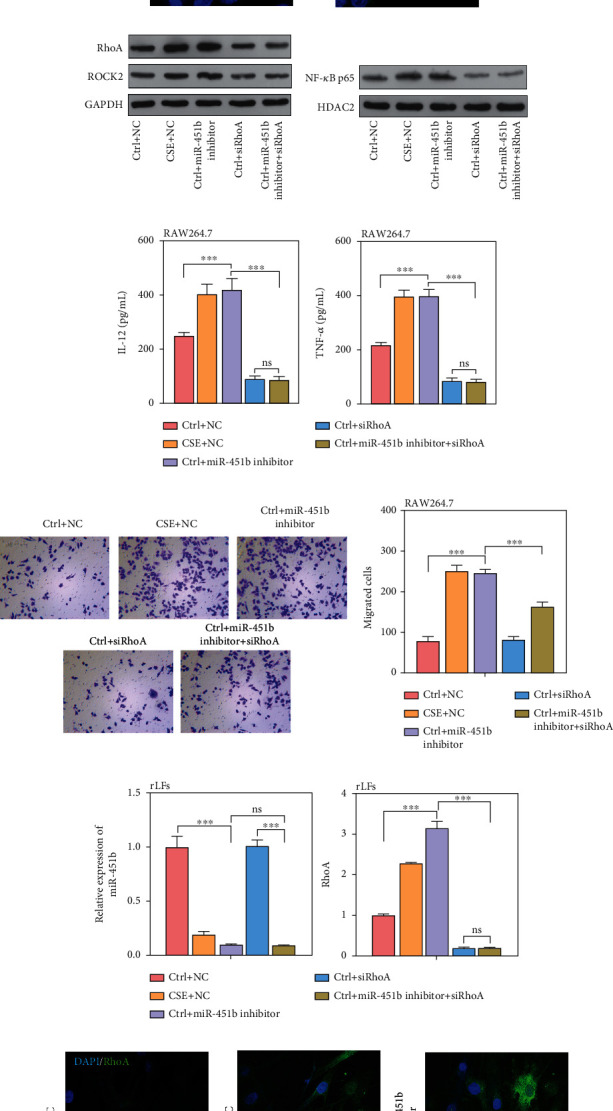
Knockdown of miR-451b activated the RhoA/ROCK2 signaling in RAW264.7 and rLFs. The RAW264.7 and rLFs were transfected with miR-451b inhibitor and specifically targeting RhoA small interference sequence. (a, g) The expression of miR-451b and RhoA mRNA was determined by QPCR. (b, h) Immunofluorescence staining demonstrated the labeling intensity of RhoA. (c, i) Western blot analysis was performed to analyze the protein expression of RhoA, ROCK2. (d) Protein level of nuclear NF-*κ*B p65 in RAW264.7. (e) IL-12 and TNF-*α* levels were detected by ELISA assay. (f) Cell migration was assessed by Transwell assay. (j) Protein level of COL1A1 and COL2A1 in rLFs. ^∗∗∗^*P* < 0.001. NC: negative control. Ctrl: no CSE treatment; CSE+ siRhoA: CSE treatment plus RhoA-knockdown; DAPI: 4′,6-diamidino-2-phenylindole; ns: no significance.

To further verify the role of miR-451b in cells and its targeted regulation to RhoA, we directly inhibited the expression of miR-451b using its inhibitor, and knock of RhoA down at the same time. Results found that both in RAW264.7 and rLFs, the expression of miR-451b was significantly downregulated by its inhibitor, while the RhoA mRNA and protein level was increased (Figures 6(a)–6(c) and 6(g)–6(i)). The protein level of ROCK2 and nuclear NF-*κ*B p65 was also increased by miR-451b inhibitor (Figures 6(c) and 6(d)). The IL-12 and TNF-*α* levels in RAW264.7 culture supernatant were significantly increased in the miR-451b inhibitor group (Figure 6(d)), as well as the cell migration ability (Figure 6), which was both reversed by knockdown of RhoA. Furthermore, the accumulation of COL1A1 and COL2A1 in rLFs was induced by miR-451b inhibitor, and which was also reversed by knockdown of RhoA, too (Figure 6(j)). These results confirm miR-451b functions through regulating RhoA.

## 4. Discussion

In the present study, we reported that miR-451b was downregulated in macrophages after CSE exposure. Overexpression of miR-451b significantly decreased the pro-inflammatory mediators (IL-12 and TNF-*α*), downregulated the expression of RohA, ROCK2, and NF-*κ*B p65, and suppressed migration and invasion ability. Macrophages are mononuclear leukocyte-derived inflammatory cells, which are correlated with the inflammatory response and alveolar wall destruction in COPD [27]. Macrophages respond to cigarette smoke by producing pro-inflammatory mediators, including IL-8, IL-12, IL-1*β*, and TNF-*α* [28]. Previous work has reported the important role of IL-12 and TNF-*α* in inflammatory airway diseases [29, 30]. In addition, macrophages are also an important source of MMP production that contributes to alveolar wall destruction [31]. Moreover, activation of RhoA/ROCK signaling could mediate macrophage differentiation induced by PMA [32]. Here, we thus selected RAW264.7 to stimulate with CSE to study the role of miR-451b on CSE-induced inflammation, migration, and invasion.

Much of the research has examined whether smoking influenced the expression of miRNAs in airway, and it is known that there is an association between differentially expressed miRNAs and COPD [33, 34]. The interactions between miRNA-mRNA-lncRNA expanded our understanding of the disease mechanism in smoking COPD [35]. As for the miR-451b in COPD pathogenesis, our previous work demonstrated that miR-451b expression was downregulated in the smoker without COPD, smoker with stable COPE, and smoker with acute exacerbation COPD groups compared with non-smoker non-COPD group [18]. Here, stimulation of CSE results in a decrease in miR-451b expression and in increase in the expression of its target gene, RhoA. Furthermore, transfection of miR-451b mimics induced the downregulation of RhoA. MiR-451b overexpression reversed the effects of CSE on macrophages. Similarly, a previous study indicated that miR-451b was associated with both childhood asthma and adult COPD exacerbations [36]. Our data further showed that increased miR-451b expression caused the alteration of RhoA/ROCK signaling and p65 protein levels. Accumulating evidence has indicated RhoA plays a crucial role on the development of COPD. For instance, CSE may impair efferocytosis through oxidant-dependent activation of RhoA [37]. CSE-induced p120-catenin- (p120-) mediated NF-*κ*B activation in human epithelial cells is dependent on the RhoA/ROCK pathway [25]. Activity of RhoA/Rho-kinase was increased in pulmonary arteries of COPD patients as compared with control subjects [22]. Furthermore, miR-133a/RhoA axis has been reported to participate in the elevation of carbon dioxide in tissues in patients with severe lung diseases, including COPD [38].

**Figure 7:**
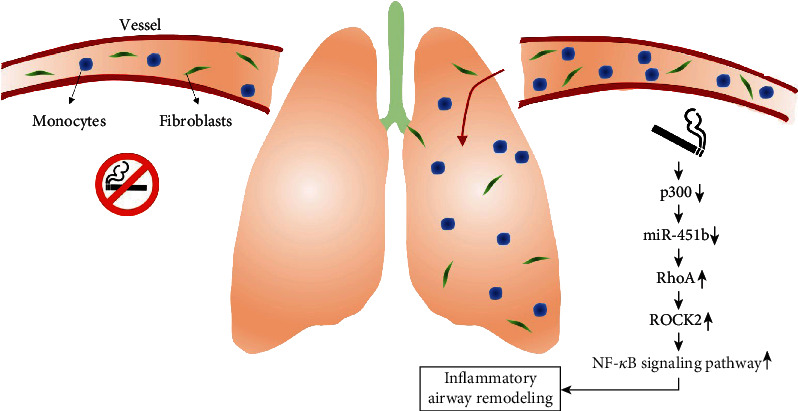
Schematic diagram of smoking-induced inflammatory and airway remodeling through regulating miR-451b.

Lung fibroblasts have been previously reported to play a significant role in orchestrating inflammatory responses and responding to cigarette smoke by increasing pro-inflammatory prostaglandins and other pro-inflammatory mediators [39]. Here, we showed that CSE exposure increased the expression of RhoA, ROCK2, COL1A1, and COL2A1 in lung fibroblasts. Importantly, miR-451b mimics transfection abolished these effects of CSE on lung fibroblasts. COL1A1 and COL2A1, as the ECM components, have been demonstrated to be the downstream of transforming protein RhoA and Rho-associated protein kinase 1 for the regulation of osteogenesis [40]. Wang et al. [41] further manifested that ECM proteins promoted proliferation, migration, and adhesion of ASMCs from rat models of COPD through activation of the PI3K/AKT signaling pathway. Based on these facts, we thus speculated that overexpression of miR-451b could attenuate the impaired functional properties of lung fibroblasts, as key players in maintaining tissue homeostasis, are believed to be an important mechanism underlying COPD (Figure 7). However, there is a lack of verification by animal experiment and which is one of the limitations in the presented study.

## 5. Conclusions

In summary, our results suggest that transcription factor p300 regulated the expression of miR-451b and the latter suppressed CSE-induced inflammation and impaired functional properties in macrophages and lung fibroblasts. These effects may be associated with the regulation of its target gene RhoA-mediated RhoA/ROCK2 signaling pathway. This study therefore identifies p300/miR-451b/RhoA axis as a potential therapeutic target for CSE-induced injury in COPD.

## Data Availability

The data that support the findings of this study are available on request from the corresponding author.

## Supplementary Materials

Supplementary Materials Table S1 Sequences of transfected substance and primers used in QPCR.

## References

[1] Barnes P. J. (2007). Chronic obstructive pulmonary disease: a growing but neglected global epidemic. *PLoS Medicine*.

[2] Disease G. B. D., Injury I., Prevalence C. (2016). Global, regional, and national incidence, prevalence, and years lived with disability for 310 diseases and injuries, 1990-2015: a systematic analysis for the Global Burden of Disease Study 2015. *Lancet*.

[3] Celli B. R., MacNee W., Force A. E. T. (2004). Standards for the diagnosis and treatment of patients with COPD: a summary of the ATS/ERS position paper. *The European Respiratory Journal*.

[4] Yang S. R., Wright J., Bauter M., Seweryniak K., Kode A., Rahman I. (2007). Sirtuin regulates cigarette smoke-induced proinflammatory mediator release via RelA/p65 NF-kappaB in macrophages in vitro and in rat lungs in vivo: implications for chronic inflammation and aging. *American journal of physiology Lung cellular and molecular physiology*.

[5] Barnes P. J. (2013). New anti-inflammatory targets for chronic obstructive pulmonary disease. *Nature Reviews Drug Discovery*.

[6] Carnevali S., Petruzzelli S., Longoni B. (2003). Cigarette smoke extract induces oxidative stress and apoptosis in human lung fibroblasts. *American journal of physiology Lung cellular and molecular physiology*.

[7] Togo S., Holz O., Liu X. (2008). Lung fibroblast repair functions in patients with chronic obstructive pulmonary disease are altered by multiple mechanisms. *American Journal of Respiratory and Critical Care Medicine*.

[8] Higham A., Bostock D., Booth G., Dungwa J. V., Singh D. (2018). The effect of electronic cigarette and tobacco smoke exposure on COPD bronchial epithelial cell inflammatory responses. *International Journal of Chronic Obstructive Pulmonary Disease*.

[9] Hulina-Tomašković A., Somborac-Bačura A., Grdić Rajković M., Bosnar M., Samaržija M., Rumora L. (2019). Effects of extracellular Hsp70 and cigarette smoke on differentiated THP-1 cells and human monocyte-derived macrophages. *Molecular Immunology*.

[10] Wang Y., Liu J., Zhou J. S. (2018). MTOR suppresses cigarette smoke-induced epithelial cell death and airway inflammation in chronic obstructive pulmonary disease. *Journal of immunology (Baltimore, Md: 1950)*.

[11] Rolle K., Piwecka M., Belter A. (2016). The sequence and structure determine the function of mature human miRNAs. *PLoS One*.

[12] Osei E. T., Florez-Sampedro L., Timens W., Postma D. S., Heijink I. H., Brandsma C. A. (2015). Unravelling the complexity of COPD by microRNAs: it’s a small world after all. *The European Respiratory Journal*.

[13] Kara M., Kirkil G., Kalemci S. (2016). Differential expression of microRNAs in chronic obstructive pulmonary disease. *Advances in clinical and experimental medicine : official organ Wroclaw Medical University*.

[14] Long Y. J., Liu X. P., Chen S. S., Zong D. D., Chen Y., Chen P. (2018). miR-34a is involved in CSE-induced apoptosis of human pulmonary microvascular endothelial cells by targeting Notch-1 receptor protein. *Respiratory Research*.

[15] Tang K., Zhao J., Xie J., Wang J. (2019). Decreased miR-29b expression is associated with airway inflammation in chronic obstructive pulmonary disease. *American journal of physiology Lung cellular and molecular physiology*.

[16] Sato T., Liu X., Nelson A. (2010). Reduced miR-146a increases prostaglandin E2 in chronic obstructive pulmonary disease fibroblasts. *American Journal of Respiratory and Critical Care Medicine*.

[17] Zhang L., Huang C., Guo Y. (2015). MicroRNA-26b modulates the NF-*κ*B pathway in alveolar macrophages by regulating PTEN. *Journal of immunology (Baltimore, Md: 1950)*.

[18] Shen W., Liu J., Zhao G. (2017). Repression of Toll-like receptor-4 by microRNA-149-3p is associated with smoking-related COPD. *International Journal of Chronic Obstructive Pulmonary Disease*.

[19] Shen W., Liu J., Fan M. (2018). MiR-3202 protects smokers from chronic obstructive pulmonary disease through inhibiting FAIM2: an _in vivo_ and _in vitro_ study. *Experimental Cell Research*.

[20] Etienne-Manneville S., Hall A. (2002). Rho GTPases in cell biology. *Nature*.

[21] Duong-Quy S., Dao P., Hua-Huy T., Guilluy C., Pacaud P., Dinh-Xuan A. T. (2011). Increased rho-kinase expression and activity and pulmonary endothelial dysfunction in smokers with normal lung function. *The European Respiratory Journal*.

[22] Bei Y., Duong-Quy S., Hua-Huy T., Dao P., Le-Dong N. N., Dinh-Xuan A. T. (2013). Activation of RhoA/Rho-kinase pathway accounts for pulmonary endothelial dysfunction in patients with chronic obstructive pulmonary disease. *Physiological Reports*.

[23] Xie X., Peng J., Chang X. (2013). Activation of RhoA/ROCK regulates NF-*κ*B signaling pathway in experimental diabetic nephropathy. *Molecular and Cellular Endocrinology*.

[24] He Y., Xu H., Liang L. (2008). Antiinflammatory effect of Rho kinase blockade via inhibition of NF-*κ*B activation in rheumatoid arthritis. *Arthritis and Rheumatism*.

[25] Zhang C., Qin S., Qin L. (2016). Cigarette smoke extract-induced p120-mediated NF-kappaB activation in human epithelial cells is dependent on the RhoA/ROCK pathway. *Scientific Reports*.

[26] Higashi T., Mai Y., Mazaki Y., Horinouchi T., Miwa S. (2016). A standardized method for the preparation of a gas phase extract of cigarette smoke. *Biological & Pharmaceutical Bulletin*.

[27] Arora S., Dev K., Agarwal B., Das P., Syed M. A. (2018). Macrophages: their role, activation and polarization in pulmonary diseases. *Immunobiology*.

[28] Yang S. R., Chida A. S., Bauter M. R. (2006). Cigarette smoke induces proinflammatory cytokine release by activation of NF-kappaB and posttranslational modifications of histone deacetylase in macrophages. *American journal of physiology Lung cellular and molecular physiology*.

[29] Bal S. M., Bernink J. H., Nagasawa M. (2016). IL-1*β*, IL-4 and IL-12 control the fate of group 2 innate lymphoid cells in human airway inflammation in the lungs. *Nature Immunology*.

[30] Shyam Prasad Shetty B., Chaya S. K., Kumar V. S. (2021). Inflammatory biomarkers interleukin 1 beta (IL-1*β*) and tumour necrosis factor alpha (TNF-*α*) are differentially elevated in tobacco smoke associated COPD and biomass smoke associated COPD. *Toxics*.

[31] Xu J., Tao B., Guo X. (2017). Macrophage-restricted Shp2 tyrosine phosphatase acts as a rheostat for MMP12 through TGF-beta activation in the prevention of age-related emphysema in mice. *Journal of immunology (Baltimore, Md: 1950)*.

[32] Yang L., Dai F., Tang L., Le Y., Yao W. (2017). Macrophage differentiation induced by PMA is mediated by activation of RhoA/ROCK signaling. *The Journal of Toxicological Sciences*.

[33] Conickx G., Avila Cobos F., van den Berge M. (2017). microRNA profiling in lung tissue and bronchoalveolar lavage of cigarette smoke-exposed mice and in COPD patients: a translational approach. *Scientific Reports*.

[34] Paul S., Ruiz-Manriquez L. M., Ambriz-Gonzalez H. (2022). Impact of smoking-induced dysregulated human miRNAs in chronic disease development and their potential use in prognostic and therapeutic purposes. *Journal of Biochemical and Molecular Toxicology*.

[35] Qian Y., Mao Z. D., Shi Y. J., Liu Z. G., Cao Q., Zhang Q. (2018). Comprehensive analysis of miRNA-mRNA-lncRNA networks in non-smoking and smoking patients with chronic obstructive pulmonary disease. *Cellular Physiology and Biochemistry*.

[36] Tiwari A., Hobbs B., Li J. (2022). Blood miRNAs are linked to frequent asthma exacerbations in childhood asthma and adult COPD. *Noncoding RNA*.

[37] Richens T. R., Linderman D. J., Horstmann S. A. (2009). Cigarette smoke impairs clearance of apoptotic cells through oxidant-dependent activation of RhoA. *American Journal of Respiratory and Critical Care Medicine*.

[38] Shigemura M., Lecuona E., Angulo M. (2018). Hypercapnia increases airway smooth muscle contractility via caspase-7-mediated miR-133a-RhoA signaling. *Science translational medicine*.

[39] Martey C. A., Pollock S. J., Turner C. K. (2004). Cigarette smoke induces cyclooxygenase-2 and microsomal prostaglandin E2 synthase in human lung fibroblasts: implications for lung inflammation and cancer. *American journal of physiology Lung cellular and molecular physiology*.

[40] Khatiwala C. B., Kim P. D., Peyton S. R., Putnam A. J. (2009). ECM compliance regulates osteogenesis by influencing MAPK signaling downstream of RhoA and ROCK. *Journal of Bone and Mineral Research: the Official Journal of the American Society for Bone and Mineral Research*.

[41] Wang Z., Li R., Zhong R. (2018). Extracellular matrix promotes proliferation, migration and adhesion of airway smooth muscle cells in a rat model of chronic obstructive pulmonary disease via upregulation of the PI3K/AKT signaling pathway. *Molecular Medicine Reports*.

