# Supplementary material for: Transcription Factor p300 Regulated miR-451b Weakens the Cigarette Smoke Extract-Induced Cellular Stress by Targeting RhoA/ROCK2 Signaling
Source: Oxid Med Cell Longev. 2022 Oct 14;2022:7056283. doi: 10.1155/2022/7056283 (PMC9586727; doi:10.1155/2022/7056283)
Supplement: Supplementary Materials — Table S1 Sequences of transfected substance and primers used in QPCR. [file 7056283.f1.docx]

Table S1 Sequences of transfected substance and primers used in QPCR.

| Name | Sequence (5’ - 3’) |
| --- | --- |
| miR-451b mimics | UGGGAGCAGCAAGAGAACGU |
| miR-451b inhibitor | ACGUUCUCUUGCUGCUCCCA |
| si- RhoA | UGUUUUCUAAACUAUCAGGGC |
| miR-451b forward | ACACTCCAGCTGGGTAGCAAGAGAACCATTAC |
| miR-451b reverse | CTCAACTGGTGTCGTGGA |
| U6 forward | CTCGCTTCGGCAGCACA |
| U6 reverse | AACGCTTCACGAATTTGCGT |
| RhoA forward | TTAGTCCACGGTCTGGTCTT |
| RhoA reverse | CCATTGCTCAGGCAACGAA |
| GAPDH forward | TGTTCGTCATGGGTGTGAAC |
| GAPDH reverse | ATGGCATGGACTGTGGTCAT |
